# Pharmacist-Led Prevention of Recurrent Cetuximab Infusion Reactions

**DOI:** 10.7759/cureus.97871

**Published:** 2025-11-26

**Authors:** Yota Yamada, Arisa Fukuyama, Motoyasu Miyazaki, Yasutaka Sumi, Haruka Fukue, Akio Nakashima, Takayuki Akasaki, Osamu Imakyure

**Affiliations:** 1 Department of Pharmacy, Fukuoka University Chikushi Hospital, Chikushino, JPN; 2 Department of Hospital Pharmacy, Faculty of Pharmaceutical Sciences, Fukuoka University, Fukuoka, JPN; 3 Department of Gastrointestinal Surgery, Fukuoka University Chikushi Hospital, Chikushino, JPN

**Keywords:** cetuximab, h2-receptor antagonist, infusion reactions, pharmacist, prolonged infusion

## Abstract

Cetuximab infusion reactions (IRs) can disrupt the continuity of effective treatment. However, there is a lack of standardized practical ward-level strategies to prevent IR recurrence. Herein, we report a single-case observation of a pharmacist-led approach combining H2-receptor antagonist premedication with infusion-time extension in a high-risk patient. A 69-year-old woman with metastatic colorectal cancer (RAS wild-type, microsatellite instability-high, BRAF V600E mutation) receiving encorafenib/binimetinib plus cetuximab developed Grade 2 IR during her first cetuximab infusion (approximately 98 min after initiation). She also exhibited hypoxemia and throat tightness. After discontinuing the infusion, she was treated with oxygen, d-chlorpheniramine, and famotidine, allowing completion at a reduced rate. For rechallenge, the interprofessional team implemented the following pharmacist-proposed optimization: routine H1 antihistamine plus 20 mg of intravenous famotidine and extension of cetuximab infusion to approximately 2 h, alongside standardized nursing surveillance. On the subsequent cycle, 465 mg of cetuximab (250 mg/m²) was administered without recurrence. No airway symptoms occurred. This pragmatic bundle targeted histamine-mediated pathways while tempering mediator release via the slower infusion, aligning with regulatory labeling that prioritizes H1 premedication and rate control yet enabling individualized risk mitigation after previous IRs. A pharmacist-driven checklist that adds H2 blockade and prolongs infusion may enhance safety and preserve treatment intensity in general ward settings where desensitization resources are limited. The generalizability of the findings is limited due to the single-patient design. Prospective validation comparing H1 alone versus combined H1 + H2 premedication, using predefined infusion-rate algorithms, is warranted.

## Introduction

Cetuximab, an epidermal growth factor receptor (EGFR) inhibitor that belongs to the monoclonal antibody class, is widely used to treat head and neck squamous cell carcinoma and in patients with RAS wild-type metastatic colorectal cancer. It interferes with signaling pathways that drive tumor cell proliferation and progression by binding to EGFR [1]. However, cetuximab infusion reactions (IRs) pose a major challenge in clinical practice, limiting its use. These reactions frequently manifest as allergy-like symptoms, including rash, pruritus, dyspnea, and in severe cases anaphylactic shock [2,3]. These adverse events not only impair the patient’s tolerability but may also necessitate treatment interruption or discontinuation, leading to a reduction in the therapeutic efficacy. In a nationwide, prospective Japanese registry (n = 2,126), overall and Grade 3-4 IRs occurred in 5.7% and 1.1% of the population, respectively, with most severe events arising within the first hour of the initial infusion [2,4]. Mechanistically, immunoglobulin E (IgE)-mediated immediate hypersensitivity and non-IgE pathways (e.g., cytokine release) are implicated in IRs [5]. As a cetuximab-specific feature, preexisting IgE to galactose-α-1,3-galactose (α-Gal) can precipitate severe reactions at the first infusion [3], a mechanism further consolidated by subsequent reviews [6]. With regard to prevention and premedication, regulatory labeling mandates H1 antihistamine premedication with strict infusion-rate control (typically 120 min for the initial 400 mg/m² dose with defined maximum rates) and specifies rate reduction/interruption for IRs and permanent discontinuation for severe events [7]. Moreover, contemporary practice guidelines further operationalize risk stratification and management workflows [8,9]. Reports from multicenter European cohorts have also highlighted regional and practice-related variability in infusion-reaction risk [10], underscoring the need for individualized risk assessment and tailored premedication strategies. In Japan, cetuximab’s package insert provides similar instructions, requiring H1 antihistamine premedication, administration of the initial infusion over at least 120 min, subsequent infusions over at least 60 min, and treatment interruption or discontinuation depending on IR severity [11]. Importantly, the recurrence risk is far from negligible. Among patients with Grade 1-2 IRs, one cohort reported a 15.7% recurrence upon rechallenge, highlighting the need to evaluate prophylactic strategies beyond standard H1 premedication [2]. In this context, the efficacy and safety of individualized measures (e.g., adding an H2-receptor antagonist and prolonging the infusion time) need to be systematically evaluated, particularly in patients with previous IRs or other high-risk features. Moreover, contemporary oncology guidance emphasizes avoiding blanket over-premedication beyond H1 antihistamines while prioritizing individualized risk assessment and infusion-rate adjustment [8]. Recognizing the histamine-mediated component of these reactions, we incorporated an H2-receptor antagonist into the standard H1 premedication to enhance symptom control in selected cases while adhering to guidelines that discourage blanket over-premedication [8,9]. Although various risk-stratified approaches and formal desensitization protocols have been described, structured, pharmacist-led protocols that combine H2 premedication with predefined infusion-time extension after a prior cetuximab IR remain scarcely reported. In the present study, we report a case wherein a pharmacist-led strategy (H2 premedication plus infusion-time extension) successfully prevented recurrent IR during cetuximab therapy. Moreover, we discuss the practical and multidisciplinary considerations for safe rechallenge.

## Case presentation

We first outline the index event and its acute management. Then, we describe a pragmatic optimization and role delineation that enabled a safe rechallenge. The patient was a 69-year-old Japanese woman (height, 164.3 cm; weight, 77.5 kg; body surface area, 1.86 m²). She had a history of type 2 diabetes mellitus, dyslipidemia, well-controlled bronchial asthma (last attack in her 40s), and osteoporosis. Her chronic medications included rosuvastatin, eldecalcitol, raloxifene, and insulin formulations. The index malignancy was ascending colon cancer with ovarian invasion and liver metastases (sT4bN3M1a, Stage IVa). Genetic testing showed microsatellite instability-high, RAS wild-type, and BRAF p.V600E mutation. Initially, she received chemotherapy with capecitabine plus oxaliplatin. Bevacizumab was added in June 2024. After six cycles, the disease was assessed as stable disease in October 2024, at which point therapy was switched to pembrolizumab. After four cycles, she underwent laparoscopic ileocecal resection and stoma closure in February 2025. In June 2025, targeted therapy with encorafenib (300 mg/day) plus binimetinib (90 mg/day) and cetuximab (initial dose, 740 mg; loading dose, 400 mg/m²; then, 250 mg/m² weekly intravenously) was initiated. During the first cetuximab infusion, the patient developed hypoxemia (peripheral oxygen saturation (SpO₂) decreased to 90%) accompanied by dyspnea (asthma-like symptoms) and throat tightness approximately 98 min after initiation. According to the Common Terminology Criteria for Adverse Events (CTCAE) v5.0 [12], the event was classified as a Grade 2 IR because the infusion was interrupted and therapeutic intervention was required. The ward nurse promptly notified the attending physician and consulted the pharmacist regarding management at the time of index IR. The ward nurse confirmed desaturation on continuous pulse oximetry. As there was no hospital-wide IR protocol, the pharmacist recommended immediate infusion interruption and pharmacologic treatment. The attending physician promptly issued orders, including supplemental oxygen at 2 L/min and 5 mg of intravenous d-chlorpheniramine plus 20 mg of famotidine, which were administered by the nursing staff. The patient’s symptoms improved promptly after intervention. The total duration of dyspnea and throat tightness was approximately 60 min. Detailed severity scoring, peak expiratory flow, and comprehensive quantitative lung examination metrics were not recorded at the index event. After team reassessment, the infusion was restarted at a reduced rate of 150 mL/h and completed under close bedside monitoring. The infusion was then completed without further instability. The patients’ vital signs are detailed in Table 1. 

**Table 1:** Vital sign changes, respiratory findings, and interventions during and after the first cetuximab infusion BP: blood pressure, HR: heart rate, RR: respiratory rate, Temp: temperature, SpO₂: peripheral oxygen saturation, RA: room air, NC: nasal cannula, IV: intravenous, NR: not recorded.

Timepoint (relative time)	BP (mmHg)	HR (bpm)	RR (breaths/ min)	Temp (°C)	SpO_2_ (%)	O_2_	Respiratory findings	Interventions/outcome
Baseline (0 min)	110/56	66	16	36.9	97	RA	None	None
Onset of reaction (98 min)	143/74	79	19	36.7	90	RA → 2L/min NC	Dyspnea + throat tightness	Infusion stopped
+20 min (120 min)	142/69	67	22	NR	97	2 L/min NC	Dyspnea improvement; persistent throat tightness	d‑Chlorpheniramine 5 mg IV + famotidine 20 mg IV
+30 min (134 min)	135/61	69	21	36.7	96	2 L/min NC	Dyspnea resolution; throat tightness improvement	Infusion resumed at 150 mL/h
+60 min (160 min)	NR	NR	NR	NR	NR	2 L/min NC	Throat tightness resolution; no recurrence	Monitoring continued
End of infusion (185 min)	138/68	79	20	36.8	99	2 L/min NC	No recurrence	Completed
60 min after end (241 min)	132/69	72	18	36.7	97	RA	No recurrence	O₂ discontinued; stable course

**Figure 1:**
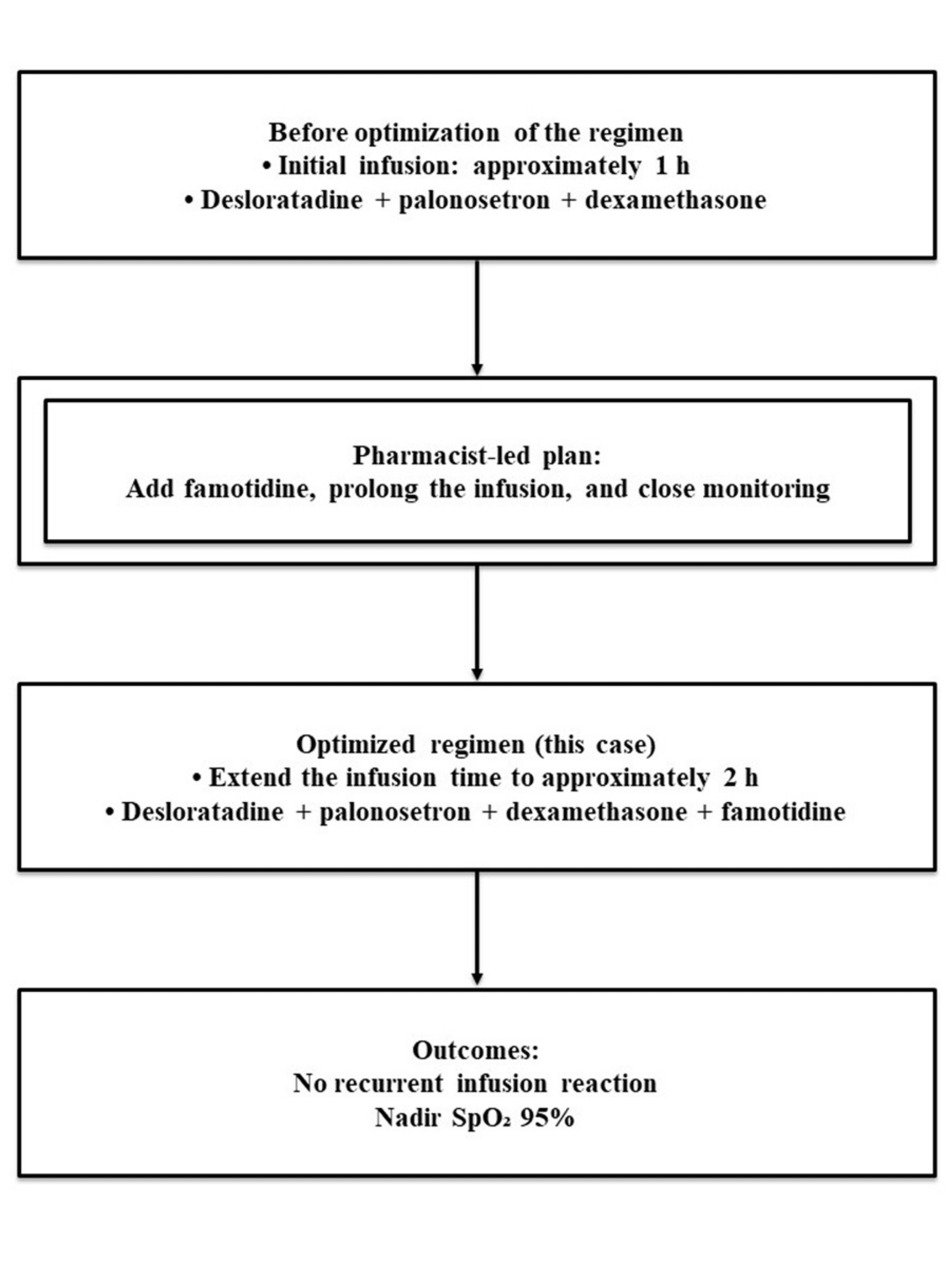
Pharmacist-led optimization of cetuximab infusion to prevent recurrence of infusion reactions

The team adopted a pragmatic bundle focusing on premedication and infusion-rate control because formal desensitization was not immediately feasible in our general ward setting. Considering the recurrence risk, the interprofessional team in the gastrointestinal surgery ward agreed on a pharmacist-proposed regimen optimization: (1) addition of 20 mg of intravenous famotidine as routine premedication in addition to label-concordant H1 antihistamine and (2) extension of the cetuximab infusion to approximately 2 h (previously 1 h). The optimized premedication and infusion plan, which was discussed with the patient as part of shared decision-making and implemented at the subsequent cycle, is summarized in Figure 1. 

The pharmacist prepared the medications and checked for interactions/contraindications, whereas the nursing staff performed patient monitoring (e.g., SpO₂, breath sounds, and symptom checks) and documentation. For the subsequent cetuximab infusion, premedication consisted of an H1 antihistamine (5 mg of desloratadine orally) administered 30 min prior, followed by intravenous palonosetron (0.75 mg), dexamethasone (9.9 mg), and an H2-receptor antagonist (20 mg of famotidine) administered 15 min before infusion initiation. Approximately 465 mg of cetuximab (250 mg/m²) was infused over 2 h with continuous monitoring. No IR recurrence was observed. Moreover, no hypoxemia or airway symptoms were noted, and the nadir SpO₂ remained at 95%. The ward maintained readiness for escalation (e.g., bronchodilator therapy or transfer for respiratory support). However, no additional interventions were required. We adopted a responsibility-assignment approach to standardize who does what, when, and with whom. Specifically, we used a concise Responsible, Accountable, Consulted, and Informed framework [13,14]. The assignments are detailed in Table 2.

**Table 2:** RACI matrix for the multidisciplinary management of cetuximab infusion reactions and safe rechallenge RACI: Responsible, Accountable, Consulted, Informed. Roles: ward nurse, pharmacist, and attending physician. Rules: one A per task and at least one R; C = two-way; I = one-way.

Tasks	Ward nurse	Pharmacist	Attending physician
Detect hypoxemia/symptoms and notify the team	R/A	C	C
Recommend treatment interruption and pharmacologic treatment	C	R	A
Issue orders (e.g., supplemental oxygen at 2 L/min; 5 mg of intravenous d-chlorpheniramine + 20 mg of famotidine)	I	C	R/A
Restart infusion at a reduced rate (150 mL/h) after team reassessment	R	C	A
Optimize treatment regimen: add H2 premedication and extend the infusion to approximately 2 h	I	R	A
Subsequent infusion: continuous monitoring and documentation	R/A	C	I

## Discussion

The present case suggests the usefulness of a pragmatic, pharmacist-led strategy combining an H2-receptor antagonist as premedication with extension of the infusion time to prevent cetuximab-associated IR recurrence. The patient was an older adult with metastatic colorectal cancer and allergic asthma who was treated in a general ward setting. The index reaction (with hypoxemia (SpO₂: 90%), dyspnea, and throat tightness) occurred approximately 98 min after the initiation of cetuximab infusion. The infusion was interrupted, and pharmacologic treatment was given, meeting the CTCAE v5.0 criteria for Grade 2 IR. After regimen optimization, subsequent infusions were well tolerated without recurrent hypoxemia or airway symptoms, enabling uninterrupted continuation of therapy. The patient completed seven once-weekly administrations between June 5 and July 31, 2025, with no recurrent IRs or dose delays, or treatment interruptions, a pattern we defined as “no recurrence.” Mechanistically, cetuximab-related IRs can arise via two principal pathways: (1) IgE-mediated immediate hypersensitivity involving preexisting α-Gal-specific IgE and (2) non-IgE-mediated cytokine release [3,5]. Patients with allergic comorbidities (e.g., asthma) are particularly vulnerable to respiratory compromise during IRs, requiring proactive risk stratification and close monitoring [8,15]. These factors (i.e., the presence of dual mechanisms and increased vulnerability of at-risk patients) help explain why reactions cluster at the first infusion and support, in selected cases, broadening the histamine-pathway coverage beyond H1 blockade (e.g., the addition of H2-receptor antagonists) and extending the infusion time to modulate mediator release [2,5,8]. The absence of recurrent IRs on rechallenge could be partly explained by tolerance development or spontaneous resolution after the index event; thus, the favorable course cannot be attributed solely to H2-receptor antagonism or infusion-time extension. In this case, despite long-standing quiescence, the index reaction involved hypoxemia (SpO₂ = 90%) accompanied by respiratory distress (dyspnea) and throat tightness, consistent with asthma-like symptoms. In anticipation of psychological burden and anticipatory anxiety regarding subsequent cycles, a pragmatic, low-complexity mitigation strategy was selected over a standard rechallenge approach, with readiness to escalate care if clinically indicated. Given the non-trivial recurrence risk following Grade 1-2 IRs [2], along with the patient’s respiratory profile and asthma history, this approach balanced feasibility with patient safety and clinical acceptability. The addition of an H2-receptor antagonist to standard H1 premedication can broaden symptom control because histamine-mediated manifestations involve H1 and H2 receptors. The major anaphylaxis guidelines position H2-receptor antagonists as adjunctive therapy, and successful rechallenge with famotidine-augmented premedication plus a slower infusion has been reported in the case of cetuximab-associated IR [15-17]. In the present case, the H2-receptor antagonist was intended to complement H1 antagonism for hemodynamic or bronchospastic features [16], whereas the extension of the infusion time aimed to decrease the peak infusion rate and modulate mediator release [18]. In addition, evidence suggests that infusion rates can influence IR risk. For some agents (e.g., rituximab), higher infusion rates were correlated with more IRs, whereas accelerated timing may be feasible for other agents under defined protocols [18]. Our approach is consistent with regulatory labeling, which emphasizes H1 premedication and infusion-rate control, and with contemporary oncology practice guidelines, which discourage routine blanket multidrug premedication while encouraging individualized risk mitigation in patients with previous IRs or other high-risk features [1,7,8,11]. Consistent with labeling, rechallenge strategies are limited to Grade 1-2 IRs; after a severe (Grade ≥ 3) infusion reaction, cetuximab should be discontinued and not re-administered. Consistent with guidance discouraging indiscriminate multidrug premedication, H2 blockade in the present case was used selectively as an adjunct to H1 antihistamine rather than as part of routine, blanket premedication. According to a previous study, 15.7% of patients with Grade 1-2 IRs experienced IR recurrence on rechallenge, arguing for proactive, patient-specific prevention rather than passive observation [2]. In this context, H2-receptor antagonist co-administration and infusion-time extension are simple and readily implementable measures that may be considered before high-resource interventions, including formal desensitization [19]. In our context, adding an H2-receptor antagonist imposed minimal direct drug cost (intravenous famotidine; US $0.70) and negligible pharmacy workflow burden, whereas extending the infusion time increases nursing workload and chair/bed occupancy. Importantly, beginning with the fourth course, the patient transitioned to receiving treatment in the outpatient chemotherapy suite. The longer infusion was accommodated within the routine outpatient chair schedule, and no dose delays occurred. Given that institutional costs and operational capacity vary, this description reflects our setting rather than serving as a universal recommendation. By contrast, formal desensitization typically requires protocolized multi-step preparation, trained personnel, and monitored settings, making it resource-intensive [8,15,19]. While rapid drug desensitization is effective, it is resource-intensive, requiring a continuously monitored setting with immediate resuscitation capability, an allergy-led multidisciplinary team, and standardized multi-bag, 12-step protocols involving sterile multi-dilution compounding and programmable pumps, which are emphasized in contemporary guidance [8,19]. Our institution does not consistently have the specialized infrastructure required; therefore, desensitization was not adopted in this case. Desensitization would be considered for more severe presentations, such as when IgE-mediated anaphylaxis is strongly suspected, or when symptoms recur despite optimized baseline measures (H1 premedication and strict infusion-rate control), provided institutional capability exists [8,19]. Another strength of this case report is the multidisciplinary collaboration that was maintained throughout the patient’s hospitalization. Ward nurses detected abnormalities early and rapidly notified the attending physician and pharmacist. The pharmacist proposed and communicated the initial measures (i.e., infusion interruption, intravenous d-chlorpheniramine, and intravenous famotidine), whereas the attending physician promptly issued bedside orders and oversaw the implementation. Thereafter, the pharmacist redesigned the premedication and infusion parameters (addition of H2-receptor antagonist plus infusion-time extension to 2 h). The nurses performed monitoring and documentation. Moreover, the physician provided final approval and integrative decision-making. This team-based model not only reduced the recurrence risk but also supported maintenance of dose intensity and treatment schedule. In this context, patients most likely to benefit include those with prior Grade 1-2 IRs, those with allergic comorbidities (e.g., asthma), and those with a negative or unknown α-Gal status, especially when maintaining dose intensity is critical and desensitization is not readily available in general ward or outpatient settings. By contrast, this approach is not recommended following prior Grade ≥ 3 infusion reactions, in the presence of recurrent IRs despite optimized H1 premedication and strict rate control, or in cases where IgE-mediated anaphylaxis is strongly suspected; in these circumstances, desensitization or drug discontinuation should be prioritized in accordance with established guidelines. Overall, the practical upper limit for this strategy is Grade 2; beyond that, desensitization is preferred when feasible. However, this study has several limitations that need to be considered. First, this is a single-patient observation without rechallenge under alternative strategies (e.g., H1 premedication alone with strict rate control), limiting causal attribution to any one component. Although we documented a total duration of dyspnea and throat tightness of approximately 60 min, standardized respiratory severity metrics (e.g., peak expiratory flow, wheeze score) were not obtained. This limits comparability across cases, and this limitation should be addressed in future reports. Second, α-Gal-specific IgE was not measured. Therefore, the index reaction’s pathophysiology remains presumptive. Moreover, available data suggest that measuring α-Gal-specific IgE before treatment may help identify high-risk patients and inform premedication and infusion planning [3,20]. Similarly, a history of allergic comorbidities (e.g., asthma) may also contribute to risk stratification. Therefore, these background factors should be incorporated into future prospective protocols. Third, a graded desensitization protocol was not employed. Although desensitization can permit continuation after severe reactions, it requires standardized protocols, trained staff, and appropriate monitoring environments, which may not be available in all centers [8,19]. Operational feasibility and costs are institution-dependent, limiting the generalizability of this approach. At our center, desensitization could not be routinely implemented due to infrastructure constraints. In addition, spontaneous resolution or tolerance development cannot be excluded, rendering causal attribution for the observed success provisional. Rechallenge following Grade 1-2 cetuximab IRs should be guided by a structured framework: confirm reaction severity and key clinical features; perform risk stratification based on allergic comorbidities, phenotype/timing, and α-Gal-specific IgE if available; optimize baseline management with H1 premedication and strict infusion-rate control; consider selective H2 co-administration plus infusion-time extension under close monitoring when residual risk or respiratory features persist; and escalate to desensitization protocols for more severe presentations or recurrence despite these measures, contingent on institutional capability [8,19]. The present case suggests that a pharmacist-led combination of H2-receptor antagonist premedication and infusion-time extension is a feasible strategy for preventing cetuximab-related IR recurrence. It may be particularly useful in settings where the use of alternative EGFR antibodies is difficult or associated with comparable risks. Future work should include prospective, standardized protocols comparing H1 premedication versus H1 plus H2-receptor antagonist premedication with predefined infusion-rate algorithms; risk biomarkers (e.g., α-Gal-specific IgE); and clearly defined outcomes. Reaffirming H1 premedication and strict rate control, selectively adding H2 blockade, and extending the infusion time with close monitoring (implemented via a pharmacist-driven checklist) represent a pragmatic, low-resource strategy suitable for general ward and outpatient settings. This approach warrants prospective validation in patients with prior Grade 1-2 cetuximab IRs.

## Conclusions

This single-case observation suggests that a pharmacist-led, checklist-driven strategy (adding an H2-receptor antagonist to label-concordant H1 premedication and extending the cetuximab infusion time to approximately 2 h) might help prevent the recurrence of Grade 1-2 IR, particularly in patients with allergic comorbidities. This low-resource approach warrants consideration in general wards and outpatient settings prior to initiating formal desensitization. However, the generalizability of these findings is inherently limited by the single-case design. Prospective protocols are needed to confirm the safety and effectiveness of this strategy, comparing H1 premedication alone versus H1 plus H2-receptor antagonist under predefined infusion-rate algorithms. These protocols should incorporate risk stratification, including α-Gal-specific IgE and history of allergic comorbidities, along with explicit eligibility criteria (e.g., prior Grade 1-2 IRs, relevant comorbidities) and standardized safety monitoring procedures with prespecified stop rules.
